# Inflammation/Coagulopathy/Immunology Responsive Index Predicts Poor COVID-19 Prognosis

**DOI:** 10.3389/fcimb.2022.807332

**Published:** 2022-03-04

**Authors:** Hui An, Jitai Zhang, Ting Li, Yuxin Hu, Qian Wang, Chengshui Chen, Binyu Ying, Shengwei Jin, Ming Li

**Affiliations:** ^1^ The Second Affiliated Hospital and Yuying Children’s Hospital of Wenzhou Medical University, Zhejiang, China; ^2^ School of Basic Medical Science, Wenzhou Medical University, Wenzhou, China; ^3^ Department of Pulmonary and Critical Care Medicine, The First Affiliated Hospital of Wenzhou Medical University, Wenzhou, China; ^4^ Department of Critical Care Medicine, The Second Affiliated Hospital and Yuying Children’s Hospital of Wenzhou Medical University, Wenzhou, China

**Keywords:** COVID-19, inflammation/coagulopathy/Immunology responsive index, predict, moderate, severe, critical patient

## Abstract

In the early stage of coronavirus disease 2019 (COVID-19), most cases are identified as mild or moderate illnesses. Approximately 20% of hospitalised patients become severe or critical at the middle or late stage of the disease. The predictors and risk factors for prognosis in those with mild or moderate disease remain to be determined. Of 694 patients with COVID-19, 231 patients with mild or moderate disease, who were hospitalised at 10 hospitals in Wenzhou and nearby counties in China, were enrolled in this retrospective study from 17 January to 20 March 2020. The outcomes of these patients included progression from mild/moderate illness to severe or critical conditions. Among the 231 patients, 49 (21.2%) had a poor prognosis in the hospital. Multivariate logistic regression analysis showed that higher inflammation/coagulopathy/immunology responsive index (ICIRI=[c-reactive protein × fibrinogen × D-dimer]/CD8 T cell count) on admission (OR=345.151, 95% CI=23.014−5176.318) was associated with increased odds ratios for poor prognosis. The area under the receiver operating characteristic curve for ICIRI predicting severe and critical condition progression was 0.65 (95% CI=0.519−0.782) and 0.80 (95% CI=0.647−0.954), with cut-off values of 870.83 and 535.44, respectively. Conversely, age, sex, comorbidity, neutrophil/lymphocyte ratio, CD8 T cell count, and c-reactive protein, fibrinogen, and D-dimer levels alone at admission were not good predictors of poor prognosis in patients with mild or moderate COVID-19. At admission, a novel index, ICIRI, tends to be the most promising predictor of COVID-19 progression from mild or moderate illness to severe or critical conditions.

## 1 Introduction

Severe acute respiratory syndrome coronavirus 2 (SARS-CoV-2) continues to spread globally. Coronavirus disease 2019 (COVID-19) is generally categorised as mild, moderate, severe, and critical (He et al., 2020; Wu and McGoogan, 2020; Jin et al., 2021a). The majority of patients (~81%) experience mild or moderate symptoms (Zhou et al., 2020), while the other patients experience severe (14%) and critical (5%) symptoms (Sun et al., 2020). This is consistent with the finding that approximately 20% of hospitalised patients with COVID-19 require admission in the intensive care unit (ICU) (Rodriguez-Morales et al., 2020; Yang et al., 2020). Patients with critical COVID-19 usually experience worsening mild or moderate illness from the 7th–14th day of the disease course, developing severe pneumonia and acute respiratory distress syndrome.

Thus, identifying early biomarkers of disease severity on admission could expedite the early intensified therapy to reduce mortality. However, there are few reports of the prognostic capacity of early biomarkers of the disease, and the information in published studies are limited to age, sex (Jin et al., 2021a), neutrophil-to-lymphocyte ratio (NLR) (Yang et al., 2020), immunological cell defects (Du et al., 2020; Jin et al., 2021a), systemic inflammation response index; liver, heart, and kidney injuries, coagulation defects, and aggregate index of systemic inflammation in predicting mortality in COVID-19 patients (Bhargava et al., 2020; Fois et al., 2020; Huang et al., 2020; Bahardoust et al., 2021; Gallo Marin et al., 2021; Hariyanto et al., 2021). However, inflammatory responses and indicators to the microorganism-induced infection are not specific for COVID-19, leading to an uncertain diagnosis.

Previously, we found that C-reactive protein (CRP, a sensitive inflammatory marker), fibrinogen, D-dimer (a coagulopathy marker), and CD8 T cell count (CD8, an immunological marker) were associated with the progression of COVID-19 (An et al., 2021; Jin et al., 2021a; Jin et al., 2021b). In this study, we sought to apply the combination of these parameters [inflammation/coagulopathy/immunology responsive index (ICIRI)] to predict poor COVID-19 prognosis at the time of admission.

## 2 Methods

### 2.1 Study Design and Participants

#### 2.1.1 Ethics Statement

The present study was conducted in accordance with the ethical guidelines of the 1975 Declaration of Helsinki and was approved by the Ethics Committee of Wenzhou Medical University (Ref 2020002). Given the urgency of the COVID-19 pandemic and global health concerns, the requirement of informed consent was waived by the Ethics Committee of Wenzhou Medical University.

This multicentre observational study retrospectively studied 694 COVID-19 patients admitted to 10 hospitals in Wenzhou City and nearby counties in Zhejiang Province, China, from 17 January 2020 to 20 March 2020. COVID-19 was confirmed by reverse transcription polymerase chain reaction in all cases, as described previously (Huang et al., 2020). Patients with COVID-19 were stratified into mild (mild symptoms with no sign of pneumonia), moderate (fever, respiratory tract symptoms, and pneumonia on chest computed tomography scan), severe (respiratory distress syndrome, respiratory rates ≥30/min, finger oxygen saturation [measured after 5 minutes of rest] ≤93%, or oxygenation index [PaO2/FiO2] ≤300 mmHg), and critical (respiratory failure requiring intubation, shock, other organ failures, or admission to the ICU) illness groups (Jin et al., 2021a). Clinical and laboratory data were recorded in a dedicated electronic database. The exclusion criteria were as follows: lack of data on CD8^+^ T cell count, CRP level, fibrinogen level, or oxygenation index ≤300 mmHg at admission.

### 2.2 Data Collection and Laboratory Procedures

We collected epidemiological, demographic, clinical, laboratory, treatment, and outcome data using a standardised data collection from electronic medical records. Three physicians (CC, BY, and TL) and a researcher (SJ) checked all data, and differences in interpretation were adjudicated between the three primary reviewers.

According to the onset of the first symptoms before admission, the day-by-day data during the course of COVID-19 were collected, as described previously (An et al., 2021). Routine clinical blood examinations, including complete blood count and serum biochemical tests, were performed on the day of admission. In particular, we assessed white blood cell count, lymphocyte count, neutrophil count, CRP level, FIB level, DD level, and CD8 count as we described previously (An et al., 2021; Jin et al., 2021a; Jin et al., 2021b). Subsequently, we extrapolated combined blood cell indexes of systemic inflammation (neutrophil/lymphocyte ratio, NLR) and formulated the inflammation/coagulopathy/immunology responsive index, which was calculated as follows: ICIRI= (CRP [mg/L] × FIB [g/L] ×DD [µg/L]/CD8 T cell count [n/µL]).

### 2.3 Statistical Analysis

Results are expressed as median (interquartile range, IQR) or number (percentage), according to the data distribution. The D’Agostino & Pearson omnibus normality, Shapiro–Wilk normality, and Kolmogorov–Smirnov tests were applied to determine data distribution. The Kruskal–Wallis test, χ² test, chi-square test with Yates’ correction, or Fisher’s exact test were used for nonparametric data.

To adjust for risk factors associated with 12-day illness progression from moderate infection to severe or critical infection during admission, univariable and multivariable logistic regression models were applied. Considering the total number of prognoses in our study (n=231) and avoiding overfitting of the model, 11 variables (sex, age, CRP, fibrinogen, D-dimer, ICIRI, leukocyte count, neutrophil count, lymphocyte count, NLR, and CD8^+^ T cell count) were selected for multivariable logistic analysis, based on univariable logistic analysis results and clinical significance. Receiver operating characteristic (ROC) curves were applied to assess the potential predictive value of risk factors for in-hospital prognosis.

A P value < 0.05 was considered statistically significant. Statistical analysis was performed using SPSS (version 19.0) and GraphPad Prism (version 9.0, La Jolla, CA, USA) software.

## 3 Results

### 3.1 Clinical Characteristics of Moderate Cases on Admission

After the exclusion of 463 cases using the exclusion criteria, 231 patients, grouped by their subsequent COVID-19 severities as mild (9 patients), moderate (173 patients), severe (36 patients), and critical (13 patients), were finally enrolled. Of the 231 patients, 73 (31.60%) had one or more pre-existing diseases, such as cardiovascular disease (including hypertension), cardio-cerebrovascular disease, diabetes, respiratory disease, cancers, benign tumours, chronic liver and kidney diseases, and hypercholesterolemia. Because a previous study demonstrated that comorbidities in cases of moderate disease were not a risk factor for poor prognosis (Cheng et al., 2020), we included patients with comorbidities in our data analysis. All patients received antiviral, Chinese herbs and supportive therapies after the diagnosis was confirmed and were discharged alive.

Of 231 patients with COVID-19, 182 (77.78%) did not progress to severe disease, and 49 (21.21%) had poor in-hospital prognosis. Briefly, in 15.58% (36/231) of patients, the condition worsened and became severe, and in 5.63% (13/231) of patients, it became critical without death (**Table 1**). Previously, we provided detailed information about this COVID-19 cohort. This study reanalysed this cohort with different onset days, days 1 to 15 before admission, 12 days post-hospitalisation, and only selected parameters for the analysis, as shown in **Table 1**. The mean patient ages were 40.0 (25.0–48.0) years in the cohort of mild cases, 48.0 (37.5–56.0) years in that of moderate cases, 48.0 (40.3–56.5) years among those who subsequently had severe cases, and 63.0 (49.0–76.5) years among those who subsequently had critical cases. Compared to patients that did not experience disease progression, patients who became critically ill were significantly older (**Table 1**).

**Table 1:** Laboratory findings of patients with mild, moderate, subsequently severe, and subsequently critical COVID-19 at admission and on post-medication days 6 and 12.

	Total (n=231)	Mild case (n = 9)	Moderate case (n = 173)	severe case (n = 36)	critical case (n = 13)	P
**Age, years**	48.0 (39.0, 56.0)	40.0 (25.0, 48.0)	48.0 (37.5, 56.0)	48.0 (40.3, 56.5)	63.0 (49.0, 76.5) ^aa/b^	0.007
**Disease history**						
Health (%)	158/231 (68.4)	8/9 (88.9)	122/173 (70.5)	23/36 (63.9)	5/13 (38.5)^b^	
Comorbidities (%)	73/231 (31.6)	1/9 (11.1)	51/173 (29.5)	13/36 (36.1)	8/13 (61.5)^b^	
HP (%)	42/231 (18.2)	1/9 (11.1)	25/173 (14.5)	11/36 (30.6)^b^	5/13 (38.5)	
CCD (%)	7/231 (3.0)	0/9 (0)	4/173 (2.3)	2/36 (5.6)	1/13 (7.7)	
Diabetes (%)	24/231 (10.4)	0/9 (0)	14/173 (8.1)	6/36 (16.7)	4/13 (30.8)^b^	
HP and Diabetes (%)	15/231 (6.5)	0/9 (0)	7/173 (4.0)	4/36 (11.1)	4/13 (30.8)^bb^	
Kidney (%)	2/231 (0.9)	0/9 (0)	0/173 (0)	1/36 (2.8)	1/13 (7.7)	
Lung (%)	8/231 (3.5)	0/9 (0)	7/173 (4.0)	0/36 (0)	1/13 (7.7)	
Cancer (%)	7/231 (3.0)	0/9 (0)	4/173 (2.3)	1/36 (2.8)	2/13 (15.4)	
**Laboratory parameters**						
**C-reactive protein, mg/L**	9.8 (3.8, 29.3)	1.7 (0.5, 3.1)	9.7 (4.5, 24.5) ^aa^	11.1 (1.5, 74.3) ^a^	41.5 (12.8, 69.7) ^aaaa/b^	0.0001
> 10 mg/L	114/230 (49.6)	0/9 (0)	85/173 (49.1) ^aa^	18/36 (50.0) ^aa^	11/12 (91.7) ^aaaa/bb/c^	
**Fibrinogen, g/L**	4.1 (3.1, 5.1)	2.5 (2.4, 3.4)	4.1 (3.2, 4.9) ^aa^	3.7 (2.8, 6.2) ^a^	5.0 (3.8, 6.3) ^aaa^	0.0009
> 4 g/L	115/230 (50.0)	0/9 (0)	89/173 (51.5) ^aa^	17/35 (48.6) ^aa^	9/13 (69.2) ^aa^	
**D-dimer, µg/L**	290.0 (170.0, 500.0)	250.0 (150.0, 356.0)	280.0 (170.0, 410.0)	390.0 (130.0, 1320.0)	1170.0 (640.0, 1525.0) ^a/bbb^	0.001
> 500 µg/L	55/230 (23.9)	1/9 (11.1)	29/173 (16.8)	14/35 (40.0) ^bb^	11/13 (84.6) ^aa/bbbb/cc^	
**ICIRI**	39.2 (7.0, 256.7)	4.4 (1.2, 10.3)	35.8 (7.6, 137.7) ^a^	597.4 (5.7, 3870.0) ^aaa/b^	1588.0 (717.0, 2632.0) ^aaaa/bbb^	<0.0001
> 800	34/230 (14.8)	0/9 (0)	8/173 (4.6)	17/36 (47.2) ^aa/bbbb^	9/12 (75.0) ^aa/bbbb^	
**Oxygen index, mmHg**	453.4 (393.6, 500.0)	500.0 (472.1, 500.0)	444.0 (394.6, 495.1) ^a^	489.0 (414.3, 500.0)	372.9 (365.5, 491.1) ^a^	0.0027
< 300 mmHg	0/196 (0)	0/9 (0)	0/138 (0)	0/36 (0)	0/13 (0)	
**Leukocyte count, ×10^9^/L**	5.0 (4.0, 6.3)	5.1 (4.5, 7.3)	4.9 (4.0, 6.1)	4.8 (3.9, 6.4)	9.9 (5.5, 12.1) ^bbb/cc^	0.0011
> 10 ×10^9^/L	13/231 (5.6)	0/9 (0)	6/173 (3.5)	1/36 (2.8)	6/13 (46.2) ^a/bbbb/cc^	
**Neutrophil count, ×10^9^/L**	3.1 (2.3, 4.2)	3.2 (2.4, 4.8)	3.0 (2.2, 3.9)	3.0 (2.1, 4.1)	7.0 (3.4, 10.4) ^bb/c^	0.0074
> 6.3 ×10^9^/L	19/231 (8.2)	0/9 (0)	9/173 (5.2)	3/36 (8.3)	7/13 (53.9) ^a/bbbb/cc^	
**Lymphocyte count, ×10^9^/L**	1.3 (0.9,1.6)	1.4 (1.3, 2.4)	1.3 (1.0, 1.6)	1.3 (0.9, 1.7)	0.9 (0.5, 1.1) ^aa/b^	0.0042
< 1.1 ×10^9^/L	79/231 (34.2)	1/9 (11.1)	54/173 (31.2)	15/36 (41.7)	9/13 (69.2) ^a/b^	
**NLR**	2.4 (1.6, 3.4)	2.0 (1.7, 2.6)	2.4 (1.6, 3.3)	2.3 (1.5, 3.7)	6.3 (3.1, 21.1) ^a/bbb/cc^	0.0013
> 3.2	69/231 (29.9)	1/9 (11.1)	49/173 (28.3)	9/36 (25.0)	10/13 (76.9) ^aa/bb/cc^	
**CD8^+^ T cells/μL**	299.0 (186.0, 436.0)	327.0 (259.5, 470.2)	330.0 (224.5, 464.5)	237.5 (137.5, 335.3) ^bb^	137.0 (137.0, 150.0) ^aa/bbbb^	<0.0001
< 320/μL	122/231 (52.8)	4/9 (44.4)	82/173 (47.4)	24/36 (66.67) ^b^	12/13 (92.3) ^a/bb^	
**ALT, IU/L**	23.0 (15.0, 39.0)	22.0 (13.0,29.5)	22.0 (14.0, 35.0)	25.0 (16.0, 48.0)	23.0 (19.5, 68.0)	0.3002
Male >40 IU/L; Female >35 IU/L	52/227 (22.9)	1/9 (11.1)	35/171 (20.5)	12/34 (35.3)	4/13 (30.8)	
**AST, IU/L**	24.0 (18.0, 34.0)	20.0 (16.0,28.0)	23.0 (18.0, 31.0)	24.2 (20.0, 41.0)	44.0 (33.5, 67.5)^aa/bb^	0.0009
>40 IU/L	35/209 (16.7)	1/9 (11.1)	18/156 (11.5)	8/31 (25.8)	8/13 (61.5)^bbbb^	
**Total bilirubin, μmol/L**	10.9 (7.3, 15.5)	9.9 (6.1, 14.8)	10.7 (7.1, 15.6)	13.4 (9.2, 15.9)	10.0 (7.0, 15.0)	0.4189
**Direct Bilirubin, μmol/L**	4.1 (2.9, 5.9)	2.9 (2.6, 4.4)	4.2 (2.8, 6.0)	4.8 (3.2, 6.0)	4.0 (3.5, 6.5)	0.2444
**Post-medication day 6**						
**Oxygen index, mmHg**	369.2 (265.0, 479.0)	337.9 (320.5, 481.0)	407.0 (352.8, 499.5)	207.4 (124.1, 271) ^bbbb^	301.3 (213.5, 500.0)	<0.0001
< 300 mmHg	26/84 (31.0)	0/5 (0)	2/48 (4.2)	21/25 (84.0) ^aa/bbbb^	3/6 (50.0) ^bb^	
**Post-medication day 12**						
**Oxygen index, mmHg**	338.0 (233.2, 428.7)	371.0 (351.1, 377.3)	416.5 (337.3, 492.9)	201.5 (121.9, 282.5) ^bbbb^	281.5 (226.8, 397.5)	<0.0001
< 300 mmHg	29/77 (37.7)	0/4 (0)	0/34 (0)	23/29 (79.3) ^aa/bbbb^	6/10 (60.0) ^bbbb^	

Data are median (IQR), or n/N (%). P values were calculated by Kruskal-Wallis test, χ² test, or Fisher’s exact test, as appropriate. ICIRI: ([c-reactive protein, mg/L*Fibrinogen, g/L*D-dimer, µg/L]/CD8^+^ T cell number, n/µL); NLR, neutrophil to lymphocyte ratio; HP, hypertension; CCD, cardio-cerebrovascular disease; ALT, alanine aminotransferase; AST, aspartate aminotransferase.

^a^p values indicate a significant difference compared with mild group; ^a^p < 0.05, ^aa^ p < 0.01, ^aaa^ p < 0.001, ^aaaa^ p < 0.0001.

b p values indicate a significant difference compared with moderate group; ^b^p < 0.05, ^bb^ p < 0.01, ^bbb^ p < 0.001, ^bbbb^ p < 0.0001.

c p values indicate a significant difference compared with Subsequently severe case; ^c^p < 0.05, ^c c^p < 0.01.

### 3.2 Laboratory Data of Patients With Moderate Infection on Admission

**Figure 1:**
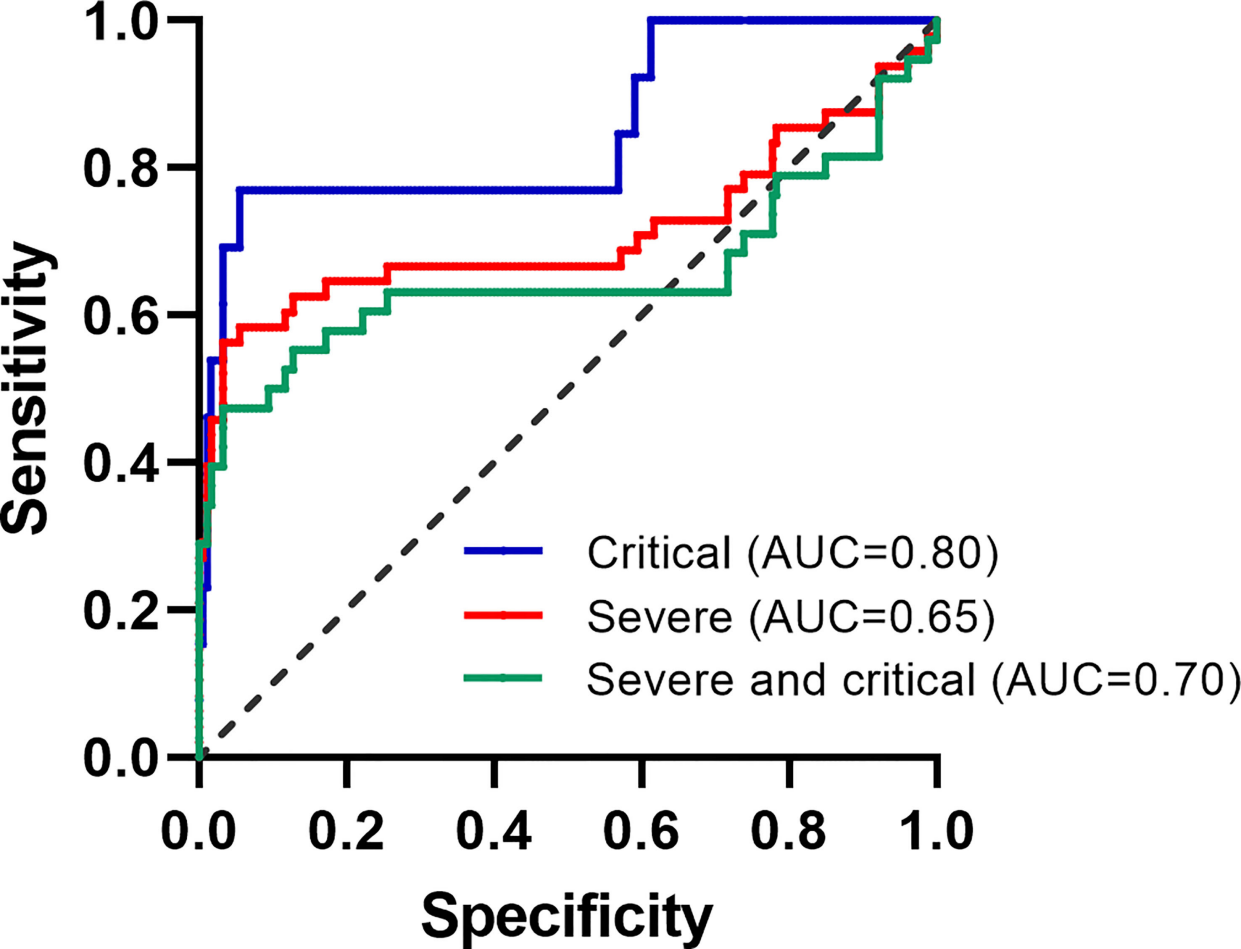
ROC curves of ICIRI in patients with moderate COVID-19.

Inflammatory and immunological variables were significantly associated with outcome, and patients with poor prognoses generally had higher levels of CRP, NLR, fibrinogen, and D-dimer, and lower lymphocyte and CD8 T cell counts (**Table 1**). Although the oxygenation index was normal in patients with subsequently critical COVID-19, CRP (41.5 [12.853–69.7] mg/L, reference: <10.00 mg/L) and D-dimer (1170.0 [640.0–1525.0] µg/L, reference: <500.0 µg/L) levels, leukocyte and neutrophil counts, and NLR (6.3 [3.1–21.1], reference: <3.2) were significantly higher in these patients than in those with moderate COVID-19. Conversely, patients with subsequently critical disease had lower lymphocyte and CD8^+^ T cell (137 [137–150] n/µL, reference >320/µL) counts than those with moderate disease at the time of admission. Plasma fibrinogen levels were significantly higher in patients with moderate (4.1 [3.2–4.9 g/L]), subsequently severe (3.7 [2.8–6.2] g/L), and critical (5.0 [3.8–6.3] g/L) COVID-19 than in those with mild (2.5 [2.4–3.4] g/L, reference <4.0 g/L) disease on admission. However, NLR >3.2 was predominant among those who subsequently had critical disease (NLR >3.2, 76.9%), but not among those who subsequently had severe disease (25.0%) when compared with those with moderate disease (28.3%) (**Table 1**). Compared to patients with moderate disease (ICIRI >800, 4.62%) at admission, higher proportions of patients with subsequently severe or critical COVID-19 had ICIRI >800 (47.22% and 75.00%, respectively, both p < 0.0001) at admission. Compared to patients with ICIRI <800 at admission, normal oxygenation index (>300 mmHg) curve, analysed *via* log-rank test, revealed that the hazard ratio of ICIRI >800 was 4.037 (95% CI=2.538–6.422, P<0.0001), after 27 days since the onset of COVID-19 symptoms (**Figure 1**). On admission, only an increase of plasma aspartate aminotransferase (AST), but not alanine aminotransferase (ALT) and bilirubin, was associated with subsequently critical patients (**Table 1**).

### 3.3 Risk Factors Associated With Poor Prognosis, Which Can Predict the Prognosis of Moderate COVID-19 Symptoms

**Table 2** summarises the results of univariable and multivariable logistic analyses of risk factors associated with progression from moderate to severe or critical conditions. After adjusting for sex, age, neutrophil count, lymphocyte count, NLR, CD8 T cell count, CRP level, fibrinogen level, D-dimer level, and ICIRI, we found that a higher ICIRI (>800) was associated with increased odds ratios for poor prognosis in both univariable (OR=26.174, 95% CI=10.661-64.261, P <0.001) and multivariable regression analyses (OR=345.151, 95% CI=23.014-5176.318, P <0.001). However, older age (>65 years), higher CRP level (>10.0 mg/L), higher D-dimer level (>500.0 µg/L), neutrophil count, lymphocyte count, and lower CD8 T cell count (<320/µL) were associated with increased odds ratios of poor prognosis in univariable regression alone. Notably, a higher AST was associated with increased odds ratios for poor prognosis in both univariable (OR=5.027, 95% CI=2.165-11.674, P <0.001) and multivariable regression analyses (OR=4.138, 95% CI=1.132-15.129, P <0.05). Among those with mild or moderate COVID-19 at admission, sex (male), higher NLR ratio (>3.2), and higher fibrinogen (>4.0 g/L) were non-significantly associated with increased odds ratios for poor prognosis in both univariable and multivariable regression analyses (**Table 2**).

**Table 2:** Risk factors associated with progression from mild or moderate to severe case.

	Univariable regression		Multivariable regression	
Demographics and clinical characteristics	OR (95% CI)	P value	OR (95% CI)	P value
**Male (vs female)**	0.789 (0.418-1.487)	0.46		
**Age, years**				
**<65**	Reference			
**≥65**	3.878 (1.741-8.641)	0.001	3.300 (0.631-17.246)	0.16
**Comorbidity**	2.086 (1.094-3.977)	0.03	2.313(0.699-7.658)	0.17
**C-reactive protein, mg/L**				
**≤10**	Reference			
**>10**	2.143 (1.124-4.083)	0.02	1.991 (0.465-8.522)	0.35
**Fibrinogen, g/L**				
**≤4**	Reference			
**>4**	1.348 (0.72-2.522)	0.35	0.281(0.056-1.398)	0.12
**D-dimer, µg/L**				
**≤500**	Reference			
**>500**	5.858 (2.972-11.544)	<0.001	0.916 (0.105-7.976)	0.94
**CD8^+^ T cells/μL**				
**<320**	2.623 (1.343-5.124)	0.005	2.975(0.905-9.777)	0.07
**≥320**	Reference			
**ICIRI**				
**≤800**	Reference			
**>800**	26.174 (10.661-64.261)	<0.001	345.151 (23.014-5176.318)	<0.001
**Neutrophil count, × 10^9^/L**	1.184 (1.037-1.351)	0.01	1.566 (1.055-2.325)	0.03
**Lymphocyte count, ×10^9^/L**	0.512 (0.274-0.958)	0.04	0.825 (0.252-2.702)	0.75
**NLR**				
**≤3.2**	Reference			
**>3.2**	1.418 (0.733-2.745)	0.3	0.504 (0.068-3.723)	0.50
**AST, IU/L**				
**<40**	Reference			
**≥40**	5.027 (2.165-11.674)	<0.001	4.138 (1.132-15.129)	0.03

OR, odds ratio; ICIRI, ([c-reactive protein, mg/L*Fibrinogen, g/L*D-dimer, µg/L]/CD8^+^ T cell number, n/µL); NLR, neutrophil to lymphocyte ratio. AST, aspartate aminotransferase.

The AUC of ICIRI for predicting total poor prognosis, progression to severe disease, and progression to critical disease were 0.704 (95% CI=0.599−0.809, P<0.001), 0.650 (95% CI=0.519−0.782, P<0.01), and 0.800 (95% CI=0.647−0.954, P<0.001), respectively, with cut-off values of 870.8, 870.8, and 535.4, respectively (**Table 3**). Other results of the ROC curve analysis, including sensitivity, specificity, Youden Index, and cut-off values, are shown in **Table 3**.

**Table 3:** The parameters results of ROC cure analysis. .

	AUC (95%CI)	P value	Sensitivity (%)	Specificity (%)	Youden Index	Cut-off value
**Prediction for total prognoses**						
**ICIRI**	0.704 (0.599-0.809)	<0.001	0.529	0.967	0.496	870.830
**Prediction for severe progression**						
**ICIRI**	0.650 (0.519-0.782)	0.004	0.486	0.967	0.453	870.830
**Prediction for critical progression**						
**ICIRI**	0.800 (0.647-0.954)	<0.001	0.733	0.906	0.639	535.441

AUC, area under the curve; ICIRI: ([c-reactive protein, mg/L*Fibrinogen, g/L*D-dimer, µg/L]/CD8^+^ T cell number, n/µL).

## 4 Discussion

In the present study, the major hepatic functional symptom of moderate COVID-19 on admission, oxygenation index, did not differ between the subsequent outcome groups. Therefore, predicting prognosis based on hepatic symptoms is challenging. Using comparative and multivariable analyses of the laboratory findings of patients with moderate COVID-19, we found that ICIRI was positively and significantly associated with poor prognosis in both patients with subsequently severe (>800: ~47%) and critical (~75%) disease when compared with those with moderately severe (~5%) disease. This suggests that among patients with moderate disease who have ICIRI >800 on admission, 5%, 47%, and 75% of cases may subsequently experience moderate, severe, and critical COVID-19, respectively. To initially differentiate mild or moderate cases from those that would subsequently become severe or critical would be helpful because it would indicate those who require close monitoring, enabling earlier treatment for patients with ICIRI >800, preventing deteriorating outcomes. In this study, the AUC of ICIRI in predicting progression to a critical condition was more than 0.80, indicating that ICIRI may act as a predictor of poor progression of COVID-19.

We also observed that higher CRP (>10.0 mg/L), higher D-dimer level (>500 µg/L), and lower CD8 T cell count (<320/µL) were accompanied by increased odds ratios of poor prognosis in univariable regression alone. Our study and others have shown that severe SARS-CoV-2 infection decreases CD8^+^ T cells (Jin et al., 2021a and Jiang et al., 2020; Wang et al., 2020). In the present study, although decrease of CD8 T cell count was accompanied by significantly increased odds ratios of poor prognosis in univariable regression alone, the multivariable regression analysis indicates that predictive relationship between severity of COVID-19 and CD8 T cell count was not reached to the significance. This might result because SARS-CoV-2 virus-induced damage to CD8 T cell count remains limited and not altered at the early stage of COVID-19. This indicates that these inflammation, coagulation, and immunology parameters alone are not strong indicators of poor COVID-19 prognosis at an early stage of COVID-19. This information is consistent with the finding of our previous study in which the use of medication after patient hospitalisation decreased inflammatory response and plasma CRP and fibrinogen levels. Medication use gradually normalised CRP concentration on post-treatment day 12 and suppressed fibrinogen levels. However, although the use of medications hindered COVID-19 progression, coagulation/fibrinolysis remained significant (An et al., 2021). This suggests that although medication use reduces the occurrence of illness due to COVID-19 progression, the antecedent thrombus resulted in an elevation in fibrinolytic activities to dissolve the preceding thrombi and gain coagulation/fibrinolysis homeostasis.

SARS-CoV-2 virus-induced overt disseminated intravascular coagulation (DIC) has been observed in patients with critical COVID-19 (Tang et al., 2020). After a viral attack, CRP activates inflammatory response and blood coagulation, impairs endogenous fibrinolytic capacity, and stimulates or enhances platelet adhesiveness and responsiveness (Chen et al., 2009). Fibrinogen has overlapping roles in blood clotting, fibrinolysis, cellular and matrix interactions, inflammatory response, wound healing, and neoplasia (Mosesson, 2005). Thus, elevated plasma CRP and fibrinogen levels could significantly influence the development or progression of the pre-DIC stage. SARS-CoV-2 not only causes damage to the lungs (Sungnak et al., 2020), endothelial cells (Varga et al., 2020), smooth muscle cells, and other cells (Meftahi et al., 2020), it also markedly decreases lymphocyte count, especially CD8 T cell count (Jin et al., 2021a). Combining our findings with other previous findings suggests that ICIRI can be a good predictor of COVID-19 progression. Further, the risk stratification of ICIRI could facilitate patient management because it suggests that patients with mild or moderate COVID-19, who have ICIRI <800 on admission, are highly unlikely to develop severe or critical illness and can be treated in a community hospital or quarantined at home. Further, the risk stratification indicates that patients with ICIRI ≥800 have a high risk of developing severe or critical diseases. They need to be prepared for transfer to a special care centre or the ICU for invasive respiratory support. We believe that by examining this concept using large-scale studies with a larger number of cases, the resultant risk stratification and management system would ease the healthcare burden associated with COVID-19 and lessen COVID-19-induced mortality.

Dissimilar to previous findings that NLR predicts the development of critical illness in COVID-19 (Cheng et al., 2020; Liu et al., 2020), we found that neutrophil count, lymphocyte count, and lower CD8 T cell count were associated with increased odds ratios for poor prognosis in univariable regression alone. Furthermore, we did not find that a higher NLR ratio was significantly associated with increased odds ratios for poor prognosis in univariable and multivariable regression analyses. This is not a surprising finding. It is well known that inflammatory responses induced by COVID-19 are influenced by both the viral infection and subsequent bacterial infections; thus, inflammatory response is not specific to COVID-19. The disparity between our data and previous findings may have been caused by the inclusion of patients at different stages of the disease in the derivation and validation cohorts, leading to an imbalance in laboratory parameters, such as leukocyte, neutrophil, and lymphocyte counts.

Previous studies show that hepatic damage marker AST is significantly higher in severe or non-survival patients with COVID-19 (Li et al., 2020; Zhang et al., 2020). In the present study, we found that only AST, but not ALT and bilirubin levels, was significantly increased in subsequently critical patients at the early stage of COVID-19. Interestingly, AST≥40 was associated with increased odds ratios for poor prognosis in univariable and multivariable regression analyses. This data suggest that AST indicator alone at an early stage of COVID-19 could be a good predictor of disease progression to critical COVID-19.

This retrospective multicentre study found that a novel index, ICIRI, at admission tends to be the most promising predictor of COVID-19 progression from mild or moderate illness to severe or critical conditions. However, this study had two main limitations. First, the study included a relatively small sample size. Second, patients with moderate COVID-19 who were enrolled in this study for prediction purposes were receiving medication (such as Chinese herbs, which have been shown to perform anti-virus, anti-inflammation, and immunoregulation *via* acting on multiple pathways to mitigate damages induced by the SARS-CoV-2 virus (Dai et al., 2020), and this may have altered their COVID-19 outcomes. Therefore, the rate of COVID-19 progression in this study may not reflect the true rate.

In summary, ICIRI seems to be the most promising predictor of progression to severe and critical COVID-19 at the time of hospital admission. AST indicator alone at the early stage of COVID-19 can be a good predictor of disease progression to critical COVID-19. Early application of these predictors and age-related prognostic data will be advantageous in patient management to prejudge cases that would become critical, requiring intensive medical care. Additionally, this would reduce the high medical care and medication burden of COVID-19. Age, sex, NLR, and CRP levels alone, measured at hospital admission, were not good predictors of a poor prognosis in patients with moderate COVID-19.

## Data Availability Statement

The original contributions presented in the study are included in the article/supplementary material. Further inquiries can be directed to the corresponding authors.

## Ethics Statement

The studies involving human participants were reviewed and approved by Ethics Committee of Wenzhou Medical University (Ref 2020002). Given the urgency of the COVID-19 pandemic and global health concerns, the requirement of informed consent was waived by the Ethics Committee of Wenzhou Medical University. Written informed consent for participation was not required for this study in accordance with the national legislation and the institutional requirements.

## Author Contributions

ML and SJ contributed equally to this study and are the joint corresponding authors. HA and JZ are joint first authors. The corresponding author conceived and designed the study. ML, SJ, HA, and JZ drafted the manuscript. ML, SJ, HA, and JZ performed data analysis, and all authors critically revised the manuscript for important intellectual content and gave final approval for the version to be published. TL, CC, YH, QW, and BY collected the data. All authors agree to be accountable for all aspects of the work in ensuring that questions related to the accuracy or integrity of any part of the work are appropriately investigated and resolved. All authors contributed to the article and approved the submitted version.

## Funding

This work was supported by the National Natural Science Foundation of China 82070855 and 81670336 (to ML), the Wenzhou Grant for Scientific Talents, Wenzhou Science and Technology Bureau RX2016003 (to ML), the Key Research and Development Program of Zhejiang Province 2019C03011 (to SJ and TL), the Special Project for Significant New Drug Research and Development in the Major National Science and Technology Projects of China 2020ZX09201002 (to TL), and the Wenzhou Science and Technology Key problem program ZY2020001 (to TL). The funders had no role in the study design, data collection, analysis, decision to publish, or preparation of the manuscript. The funder had no role in the study design, data collection, analysis, interpretation, or report writing. The corresponding authors (ML and SJ) had full access to all the data in the study and had the final responsibility for the decision to submit the manuscript for publication.

## Conflict of Interest

The authors declare that the research was conducted in the absence of any commercial or financial relationships that could be construed as a potential conflict of interest.

## Publisher’s Note

All claims expressed in this article are solely those of the authors and do not necessarily represent those of their affiliated organizations, or those of the publisher, the editors and the reviewers. Any product that may be evaluated in this article, or claim that may be made by its manufacturer, is not guaranteed or endorsed by the publisher.
